# The Resilience Function of Character Strengths in the Face of War and Protracted Conflict

**DOI:** 10.3389/fpsyg.2015.02006

**Published:** 2016-01-12

**Authors:** Anat Shoshani, Michelle Slone

**Affiliations:** ^1^Baruch Ivcher School of Psychology, Interdisciplinary Center HerzliyaHerzliya, Israel; ^2^School of Psychological Sciences, Tel Aviv UniversityTel Aviv, Israel

**Keywords:** character strengths, war, terrorism, adolescents, resilience, coping, positive-psychology, PTSD

## Abstract

This study investigated the role of character strengths and virtues in moderating relations between conflict exposure and psychiatric symptoms among 1078 adolescents aged 13–15 living in southern Israel, who were exposed to lengthy periods of war, terrorism and political conflict. Adolescents were assessed for character strengths and virtues, political violence exposure using the Political Life Events (PLE) scale, and psychiatric symptoms using the Brief Symptom Inventory and the UCLA PTSD Index. Results confirmed that political violence exposure was positively correlated with psychiatric symptoms. Interpersonal, temperance and transcendence strengths were negatively associated with psychiatric symptoms. Moderating effects of the interpersonal strengths on the relation between political violence exposure and the psychiatric and PTSD indices were confirmed. The findings extend existing knowledge about the resilience function of character strengths in exposure to protracted conflict and have important practical implications for applying strength-building practices for adolescents who grow up in war-affected environments.

## Introduction

Increasing research on exposure to political violence, terrorism and war has led to significant advances in comprehension of the nature of post-traumatic stress disorder (PTSD) and psychological responses of youth to these environments (Betancourt and Khan, 2008). However, more systematic research under the rubric of resilience indicates that many people who live in chronic war zones emerge less damaged than traditional theories might expect (Bonnano, 2004). This evidence raises questions about the characteristics that help children and adolescents exhibit posttraumatic adaptation and prevail in the aftermath of traumatic war experiences.

The tasks of adolescence, defined by intra-psychic energy expended predominantly at establishing personal identity from among alternative identities, include the establishment of self-autonomy and social role consolidation (Marcia, 1994). These developmental challenges, together with the rapid physiological, cognitive, social and emotional transitions characteristic of the period are amplified in traumatic environments such as that of armed conflict, protracted hostilities, war and terrorism (Slone and Shoshani, 2014). Exposure to armed conflict, war and political violence has been found to have damaging effects on youth, notably on posttraumatic stress (PTS) symptoms and a wide range of both overt and covert psychiatric symptoms (Betancourt and Khan, 2008).

Evidence supports the ‘dose–response’ relation between traumatic exposures and mental health such that the greater the exposure, the more psychological problems experienced (Braun-Lewensohn et al., 2009). A study examining the effects of conflict exposure over a 14 years period in Israel revealed that periods of severe conflict involving exposure to cumulative high levels of violence were associated with high prevalence of severe psychological symptoms and PTSD among adolescents as opposed to quieter periods (Slone and Shoshani, 2014). In addition, a direct relation has been found between severity and amount of personal exposure to political conflict events and psychopathology and PTSD (Shoshani and Slone, 2008a; Slone et al., 2008, 2009; Guttmann-Steinmetz et al., 2011).

However, not all adolescents experience adverse consequences in response to distress, suggesting that there are key individual difference factors underlying the relation between response to negative affect and psychopathology among adolescents (Shoshani and Slone, 2008b). Examining the ways in which adolescents cope with these dangerous environments has particular pertinence because traumatic events experienced by adolescents may shape adult growth and development (Ballard et al., 2015).

The emerging field of positive psychology provides a conceptual framework for exploring the role of human strengths in adapting to difficult life events (Peterson et al., 2008). The present study examined the role of character strengths and virtues as they relate to adaptation during chronic armed conflict. Current conceptualizations view character as a set of global personal strengths and virtues that together form a basic positive foundation of the personality (Peterson and Seligman, 2004). In 2004, a large-scale project conducted by the Values in Action (VIA) Institute traced core virtues recognized across cultures, religions and philosophical traditions throughout history as constituting 24 widely acknowledged and acclaimed character strengths (Peterson and Seligman, 2004). Findings have shown that this family of character strengths is related to good mental health, adjustment and well-being in a variety of contexts (Leontopoulou and Triliva, 2012).

However, the question of the function served by good character in coping with traumatic circumstances has not been adequately explored and the process has not been examined for youth. The current study examined the character strengths as moderators of the relation between exposure to political violence and psychiatric symptoms among Israeli adolescents in a context of political violent conflict.

### Character Strengths As Resilience Factors

The study of various functions of good character has flourished since the VIA project (Peterson and Seligman, 2004; Park and Peterson, 2006). The VIA perspective postulates character as being comprised of varying, yet relatively stable traits, that are malleable and develop differently depending on the individual’s environment (Peterson and Seligman, 2004). Peterson and Seligman (2004) drew an important distinction between possessing a single character strength and overall good character and highlighted that good character is not absolute but should take into consideration gradations when evaluating its components.

This conceptualization proposes a hierarchical classification of two positive characteristics of good character that distinguish two conceptual levels: virtues and character strengths. Virtues are perceived as acquired qualities that enable individuals to flourish or to achieve a good life (Mintz, 1996). Based on a survey of historical religious and philosophical texts, Peterson and Seligman (2004) identified six core virtues: transcendence, humanity, wisdom, temperance, courage, and justice. The universality of these virtues from an evolutionary perspective suggests that they may play a role in human survival.

Character strengths are defined as the psychological components that enable an individual to exhibit virtues (Park, 2004). The VIA identified a final set of 24 character strengths that can be detected in human thought, behavior, and emotions. This model differs from moral competence research in the emphasis on moral virtues and the tendency to behave in a moral manner rather than on the study of moral rules and value systems (Park and Peterson, 2006).

The virtue of wisdom consists of strengths associated with the ability to seek and value knowledge and the use of information and reflective judgment in the service of the good life. This virtue includes cognitive strengths such as creativity, open-mindedness, curiosity, love of learning and perspective (Peterson and Seligman, 2004). Temperance consists of strengths that guard against indulgence and excesses and involve regulating desires, aspirations, behaviors and emotions. This consists of the ability for forgiveness, prudence, humility, modesty, and self-regulation. The virtue of transcendence consists of strengths associated with seeking and valuing a higher meaning, or belief in a purpose beyond oneself such as reflected in hope, gratitude, and appreciation of beauty, humor and spirituality. Courage includes strengths involving the resolve to achieve goals when confronted with danger, challenge, risk or adversity. These strengths consist of bravery, persistence, vitality and integrity. The virtue of justice comprises civic strengths that form the basis of healthy, involved and active community life such as a good citizenship, leadership and a sense of fairness. The virtue of humanity is comprised of interpersonal strengths reflected in caring relationships including the capacity to love and to be loved, kindness and social intelligence (Peterson and Seligman, 2004). Factor analysis of the VIA Inventory produced reduction of the six virtues to four factors that have been replicated across studies: interpersonal, temperance, intellectual, and transcendence strengths (Park and Peterson, 2006; Shoshani and Slone, 2013).

Most VIA character strengths show strong correlations with components related to well-being such as self-acceptance, purpose, mastery and mental health (Leontopoulou and Triliva, 2012). In addition, character strengths in children and adolescents have been associated with desirable outcomes such as subjective well-being, social adjustment, life satisfaction, fewer symptoms of depression and suicidal ideation (Park, 2004; Shoshani and Aviv, 2012), and less social problems such as substance use, alcohol abuse and violence (Park, 2004). Character strengths have not been studied in the context of protracted political violence and war, although there is a strong rationale to suggest the resilience role of the four strength factors in these conditions.

The transcendence strengths involve framing evaluations of the same life events in a more positive manner (DeNeve and Cooper, 1998). Research evidence supports the moderating effect of positive framing of difficult circumstances. A sense of hope enables reframing of difficult circumstances by evoking expectations of positive changes and a better future (Shoshani et al., 2015). Gratitude can mitigate PTSD symptoms by promoting greater appreciation of life and reducing negative affect (Israel-Cohen et al., 2015). A study following 9/11 showed that gratitude, hope and spiritual meaning were related to better mental health, and optimism was inversely related to PTSD (Fredrickson et al., 2003). Religiousness has been found to serve as a resilience factor during war by promoting the attribution of a broader sense of meaning (Betancourt and Khan, 2008). Among war-affected children in Sri Lanka, Fernando (2007) found that resilient orphans who identified with Buddhist religious practices were better able to make sense of, and ultimately accept, the traumatic past they had survived. This array of strengths could transform negative emotions and thoughts into a more positive outlook, thereby increasing coping with the difficult circumstances.

The temperance strengths are based on emotional and behavioral self-control and regulation and serve as a guard against indulgence and excesses by regulating desires, aspirations, behaviors and emotions (Peterson and Seligman, 2004). Self-regulatory processes involve a wide spectrum of responses including attempts to initiate, alter or terminate a stressful situation, or to decrease the intensity of cognitions, emotions, and behaviors (Raver, 2004). These abilities have been strongly associated with successful coping after traumatic events because they produce shifts in attention from fear-arousing scenes and enable emotional control or distraction (Cole et al., 2004; Lengua et al., 2005). During war, children have been found to use emotional regulation strategies to achieve and maintain balance and to adapt to overwhelmingly frightening events (Punamäki et al., 2014). A study examining children exposed to the 9/11 attacks found that lack of inhibitory control was associated with higher levels of posttraumatic symptoms (Lengua et al., 2005). A study of Russian children exposed to the terrorist attack on a school in Beslan found that emotional regulation had a protective function on mental health (Moscardino et al., 2008).

The interpersonal strengths include a repertoire of social behaviors that facilitate the use of support networks and the opening of external regulatory channels that can alter experience of a stressful situation (Shoshani et al., 2014). The association between the interpersonal strengths and lower levels of symptoms has received wide research support. Offering and receiving support during exposure to war and political conflict has been shown to promote collective coping, assistance, and symptom relief (Slone and Shoshani, 2008a). In disaster situations, shared experiences with peers and social support were found to provide significant comfort (Moore and Varela, 2010). The ability to mobilize emotional support from significant others has a beneficial effect on the potential short- and long-term negative outcomes of terrorism-induced stress (Fremont, 2004). Further, promoting the utilization of social support is one of the main objectives in teaching adolescents effective coping skills in times of war (Slone et al., 2013).

Regarding the intellectual strengths, Peterson and Seligman (2004) maintained that the capacities for perspective-taking and for finding meaning and purpose play a significant role in ego resiliency in stressful environments. In the context of war and protracted conflict, adolescents’ ability to find meaning and purpose within the broad social and political context was found to function as a resilience factor (Slone and Shoshani, 2006). An intervention study conducted during a high terrorism period in Israel showed that increasing attribution of meaning and perspective-taking reduced psychological distress symptoms (Slone and Shoshani, 2008b). In addition, a study conducted after 9/11, showed that children who rationalized the events through cognitive appraisal had significantly lower levels of anxiety (Hock et al., 2004).

## The Present Study

The present study examined the moderating role of the VIA character strength factors on consequences of war exposure among adolescents living in a city in southern Israel near the Gaza Strip. This area has been exposed for a long period to high levels of political violence, including rocket attacks and military operation in the context of the Israeli – Palestinian conflict (Golan and Shai, 2004). Gaza has been devastated by a blockade on all borders and by repeated military operations and extremely violent hostilities with Israel. Israel has endured almost a decade of rocket and missile attacks, initially restricted to the south of the country, but increasingly reaching central and northern Israel. Inhabitants in the region have between 15 s to 1 min to find shelter. The present study was conducted in June 2014 in which 181 rockets and 16 mortars were fired onto southern Israel and was preceded by 2273 rockets and 214 mortars fired in 2012–2013 (Zucker and Kaplan, 2014). Since exposure in chronic conflict is cumulative, children in Israel have been exposed to a wide variety of stressful and traumatic events related to intractable conflict (Slone and Shoshani, 2014).

War exposure was operationalized with the Political Life Events (PLE) scale (Slone et al., 1998; Slone and Shechner, 2009). Consequences of PLE exposure examined were the two theoretically related constructs of general psychological distress as measured by the Global Severity Index (GSI) of the Brief Symptom Inventory (Derogatis, 2001) and the PTSD severity score as measured by the UCLA PTSD Index (Rodriguez et al., 1999).

The study advanced two hypotheses and one exploratory question. The first hypothesis predicted a main positive effect of PLEs exposure on the GSI and PTSD score. In line with previous research showing negative correlations between character strengths and mental illness, the second hypothesis predicted negative correlations between the strengths factors and GSI and PTSD symptoms. According to the presumed stress-buffering role of the strength factors, the exploratory question examined possible moderating effects of strength factors on the relation between PLEs exposure and the outcome variables.

## Materials and Methods

### Participants

Participants were 1078 eighth and ninth grade adolescents (542 girls, 536 boys) aged 12.9–15.4 (*M* = 13.73, *SD* = 0.84) from four public middle schools in the city of Ashkelon in southern Israel. The study population consisted of a majority from middle SES status (61%) and others from low SES (22%) and high SES (17%) groups. Pupils were mostly Jewish (93%), with 4% reporting Orthodox adherence, 28% traditional and 68% secular.

### Measures

#### VIA Inventory of Strengths for Youth (VIA-Y; Park and Peterson, 2006)

The 198 item questionnaire measures 24 character strengths reflecting four scales of temperance, intellectual, transcendence and interpersonal strengths, rated on a five-point Likert scale. The VIA-Y includes four subscales: intellectual strengths of creativity, curiosity, love of learning, fairness, open-mindedness, and appreciation of beauty; temperance strengths of self-regulation, authenticity, perseverance and prudence; transcendence strengths of religiousness, hope, zest, humor, love, social intelligence, forgiveness, leadership, perspective and gratitude; and interpersonal strengths of modesty, kindness, teamwork, and bravery. The VIA Youth Survey reports good psychometrics with positive correlations between parent and self-ratings of the strengths (Park and Peterson, 2006) and in the current study yielded satisfactory Cronbach’s alpha coefficients (0.78–0.85).

#### Political Life Events scale (Slone et al., 1998; Slone and Shechner, 2009)

The PLE scale contains 20 event items that participants mark for exposure over the past year. The PLE severity score is calculated by summing all items marked positive for exposure, weighted on the basis of previously determined assessments of severity according to the formula: mild items (e.g., A security drill at school) multiplied by 1, moderate items (e.g., Harm to property as a result of terrorism, political violence or rocket attacks) multiplied by 2, and severe items (e.g., Injury to a family member as a result of war or military circumstances) multiplied by 3.

The PLE scale has been widely used and has shown high predictive validity for communities in conflict areas (Slone and Shoshani, 2014) and good discriminant validity with cross-nationality transferability for Jewish and Arab Israeli youth (Lavi and Slone, 2011, 2012), for Palestinian youth (Slone et al., 1998) and for black and white South African adolescents (Slone et al., 2000). Test–retest scores have ranged from *r* = 0.86 to *r* = 0.94 (Slone and Shechner, 2009). There is empirical support for the distinctiveness of the PLE from general life events scales (Slone and Roziner, 2013). This study yielded a Cronbach’s alpha coefficient of 0.93.

#### Brief Symptom Inventory-18 (Derogatis, 2001)

The BSI-18 is an effective screen for psychological distress and psychiatric disorders. It comprises 18 self-report items rated 0–4 and yields four subscale scores: Somatization, Depression, Anxiety, and Panic, with internal consistencies ranging from 0.74 to 0.89. The GSI is considered the best indicator of depth of distress, calculated as the average of ratings assigned to all symptoms. In the current study, the BSI-18 yielded a Cronbach’s alpha coefficient of 0.86.

#### UCLA PTSD Index for DSM-IV (Rodriguez et al., 1999)

The PTSD-I adolescent version is a 22-item self-report inventory designed to evaluate PTSD symptoms among adolescents who have experienced traumatic events. Items assess the occurrence and frequency of PTSD symptoms during the past month rated on a 0–4 Likert scale. Items correspond directly with DSM IV criteria for PTSD namely, intrusion, avoidance, and arousal criteria, while two additional items assess associated features of fear of recurrence and trauma-related guilt. Summing the ratings for each item yields an overall PTSD severity score that ranges from 0 to 68. The instrument has demonstrated strong convergent validity, high internal consistency (Cronbach’s alpha ranging from 0.88 to 0.92) and a test–retest reliability coefficient of 0.84. In this study, Cronbach’s alpha coefficients of the scales ranged from 0.84 to 0.90.

### Procedure

After receiving authorization from the Israeli Ministry of Education Ethics Committee and from school principals, schools acquired written informed consent from parents and pupils. All participants gave written informed consent in accordance with the Declaration of Helsinki. There were no objections to participating in the study. Data were collected in June 2014. All participants completed the questionnaire battery in the classroom in the presence of two experimenters, post-graduate students in Psychology, who were available for assistance on request. Questionnaires were presented in randomly counter-balanced order to prevent response set. Anonymity and confidentiality were assured and respondents were allowed to terminate participation at any point.

### Data Analysis

Two sets of hierarchical regression analyses (SPSS 21) were used to test the effects of PLE exposure and the strengths factors on the GSI and PTSD symptoms. In the first hierarchical (three step) linear regression analysis, the GSI was entered as the dependent variable and in the second, PTSD symptoms were entered as the dependent variable. In both analyses, the independent variables were PLE (first block), the four character strengths factors (second block), and interaction variables of the PLE and the strengths factors (third block). In order to achieve model convergence and parsimony, we omitted non-significant interaction effects from the final models (Cohen and Cohen, 1983). Results of the regression analyses are reported, including standardized betas, *R*^2^, and change in *R*^2^ for each step in the equation. To understand the nature of significant interactions, we examined the GSI and PTSD as a function of PLE exposure and the strengths factor at 1 standard deviation above and below the mean (Cohen and Cohen, 1983).

## Results

### Preliminary Data Analyses, Descriptive Statistics and Correlations

Preliminary data analyses suggested that all variables were normally distributed with no unusual kurtosis or skewness and, therefore, no data transformations were undertaken. We used the SPSS Missing Value Analysis package to estimate the pattern of missing data and impute missing values in appropriate procedures. Missing data was less than 3% of the total data across all the study variables, and so most common methods for dealing with missing values would yield similar results (Tabachnick and Fidell, 2007). A multiple imputation method was used to replace missing values with values that draw from a distribution of possibilities of those missing values.

Means and standard deviations of the study variables are presented in **Table 1.** Comparison of the BSI-18 general severity index (GSI) in this study to the Israeli adolescent norms of the GSI (*M* = 1.40, *SD* = 0.65) (Slone and Shoshani, 2014), revealed no significant differences between the two groups. Analysis of PTSD symptom levels in the UCLA PTSD Index according to Rodriguez et al. (1999) revealed that 24.1% of the sample reported low or negligible symptom levels, 47.3% reported moderate symptom levels, and 28.6% reported severe levels of PTSD symptoms. A significant portion of the sample was exposed to mild and moderate political events, and approximately 9% of the sample were exposed to at least one severe PLE (see **Table 2**).

**Table 1:** Means and Standard Deviations for the PLE, GSI, PTSD and the VIA Strengths Factors.

	Mean	*SD*
Political life events	8.64	5.15
General severity index	1.41	0.82
PTSD	26.57	14.21
Intellectual strengths	3.41	0.48
Interpersonal strengths	3.55	0.45
Temperance strengths	3.16	0.37
Transcendence strengths	3.51	0.47


**Table 2:** Sample proportion of exposure to political life events.

PLE scale items		Frequency of exposure
Mild events	A security drill	92.4%
	A security check in a public place	80.9%
	A suspected dangerous explosive	26.9%
	Witnessing a violent demonstration	17.3%
	Exposure to acts of political violence through the media	91.6%
	An acquaintance was involved in a violent demonstration	22.4%
Moderate events	Spending time in a security shelter	93.9%


	Harm to property as a result of terrorism, or rocket attacks, or political violence	1%
	Extended absence of a family member due to political or military involvement	27.9%
	Participation in a violent demonstration	15.6%
	An acquaintance was witness to an act of political violence or terrorism	44.2%
	Injury to an acquaintance as a result of political violence or military or terrorism	16.1%
	Confiscation of a friend’s or an acquaintance’s land	1%
Severe events	Direct exposure to gunshot, missiles, or the use of other explosives	8.5%
	Victim of an act of political violence	0.09%
	Witnessing an act of political violence or terrorism	7.1%
	Death of a family member as a result of military, or terrorism, or political violence	6.5%
	Death of a friend or acquaintance as a result of political violence	3.4%
	Injury to a family member as a result of military, or terrorism, or political violence	8.9%
	Confiscation of land of the family	0.03%


Bivariate correlations between age, gender, PLE, PTSD, GSI, BSI-18 subscales, and the strengths factors are presented in **Table 3.** This intercorrelation matrix revealed significant moderate positive correlations between PLE and PTSD symptoms, GSI and BSI-18 subscales, and negative correlations between the temperance, transcendence and interpersonal strengths and several psychiatric indices. Age and Gender (coded as 1 = female, 0 = male) showed no significant correlations with the overall GSI and PTSD scores. T-test analyses were used to assess potential gender differences in the above variables. These revealed that girls had higher levels of somatic symptoms (*M* = 1.58, *SD* = 1.01) than boys (*M* = 1.32, *SD* = 0.79), *t*(1076) = 4.70, *p* < 0.001. Additionally, girls had higher levels of interpersonal strengths (*M* = 3.61, *SD* = 0.37) than boys (*M* = 3.42, *SD* = 0.39), *t*(1076) = 8.21, *p* < 0.001. No significant gender differences were found in the other variables.

**Table 3:** Bivariate correlations between age, gender, political life events, psychological symptoms, and character strengths factors.

Variable	1	2	3	4	5	6	7	8	9	10	11	12	13
(1) Age	-												
(2) Gender	-0.01	-											
(3) PLE	-0.01	0.02	-										
(4) PTSD	-0.02	0.03	0.38ˆ***	-									
(5) BSI GSI	0.04	0.05	0.36ˆ***	0.44ˆ***	-								
(6) BSI somatization	0.01	0.07ˆ*	0.28ˆ***	0.42ˆ***	0.49ˆ***	-							
(7) BSI depression	0.08ˆ*	0.04	0.22ˆ***	0.37ˆ***	0.39ˆ***	0.52ˆ***	-						
(8) BSI anxiety	0.03	0.03	0.24ˆ***	0.35ˆ***	0.54ˆ***	0.53ˆ***	0.46ˆ***	-					
(9) BSI panic	0.01	0.01	0.19ˆ***	0.36ˆ***	0.54ˆ***	0.51ˆ***	0.51ˆ***	0.51ˆ***	-				
(10) Intellectual strengths	-0.07	0.05	0.06	0.01	0.14ˆ***	0.07ˆ*	0.06	0.04	0.05	-			
(11) Interpersonal strengths	-0.02	0.08ˆ*	0.04	0.03	-0.08ˆ*	-0.10ˆ**	-0.03	0.02	0.05	0.15ˆ***	-		
(12) Temperance strengths	0.07ˆ*	-0.05	-0.03	-0.09ˆ**	-0.13ˆ***	-0.11ˆ**	-0.11ˆ**	-0.09ˆ*	-0.11ˆ**	-0.01	0.06ˆ*	-	
(13) Transcendence strengths	-0.08ˆ*	0.03	0.06	0.03	-0.15ˆ***	-0.03	-0.16ˆ***	-0.06	0.01	0.17ˆ***	0.12ˆ***	0.06ˆ*	-


*PLE, political life events; BSI GSI, general severity index of the Brief Symptom Inventory; *^∗^p* < 0.05, ^∗∗^*p* < 0.01, ^∗∗∗^*p* < 0.001.*

### Prediction of GSI by PLE Exposure and Character Strengths

For the GSI analysis, the linear combination of the predictors was found to be significantly related to GSI levels, *R* = 0.40, *R*^2^ = 0.16, *F*(6,1071) = 27.12, *p* < 0.001. As expected, PLE exposure (*b* = 0.36, *SE* = 0.03, *t* = 10.67, *p* < 0.001) was significantly and positively correlated with GSI levels. In addition, the temperance (*b* = -0.13, *SE* = 0.03, *t* = 3.84, *p* < 0.001), transcendence (*b* = -0.16, *SE* = 0.04, *t* = 4.11, *p* < 0.001), and interpersonal strengths (*b* = -0.08, *SE* = 0.04, *t* = 2.34, *p* = 0.02) were negatively correlated with GSI levels, while the intellectual strengths (*b* = 0.14, *SE* = 0.03, *t* = 3.84, *p* < 0.001) were positively correlated with the GSI as shown in **Table 4.**

**Table 4:** Summary of hierarchical multiple regression examining predictors of general severity index (GSI) and PTSD symptoms.

	GSI				PTSD symptoms			
		
Model	β	*P*-value	Partial *r*	*R*^2^ change	*β*	*P*-value	Partial *r*	*R*^2^ change
**Step 1**								
(Constant)	0.05				0.01			
PLE	0.36ˆ***	0.000	0.35	0.09	0.39ˆ***	0.000	0.37	0.13
**Step 2**								
Intellectual strengths	0.14ˆ***	0.000	0.14	0.05	0.01	0.69	0.01	0.01
Interpersonal strengths	-0.08ˆ*	0.02	-0.08		0.02	0.41	0.02	
Temperance strengths	-0.13ˆ***	0.000	-0.14		-0.09ˆ**	0.006	-0.09	
Transcendence strengths	-0.16ˆ***	0.000	-0.14		0.03	0.38	0.03	
**Step 3**								
PLE ^∗^ Interpersonal strengths	-0.11ˆ***	0.000	-0.14	0.02	-0.09ˆ**	0.007	-0.09	0.01


*For GSI: *R* = 0.40, *R*^2^ = 0.16, *F*(6,1071) = 27.12, *p* < 0.001; For PTSD symptoms: *R* = 0.39, *R*^2^ = 0.15, *F*(6,1071) = 25.91; *p* < 0.001. ^∗^*p* < 0.05, ^∗∗^*p* < 0.01, ^∗∗∗^*p* < 0.001.*

**FIGURE 1:**
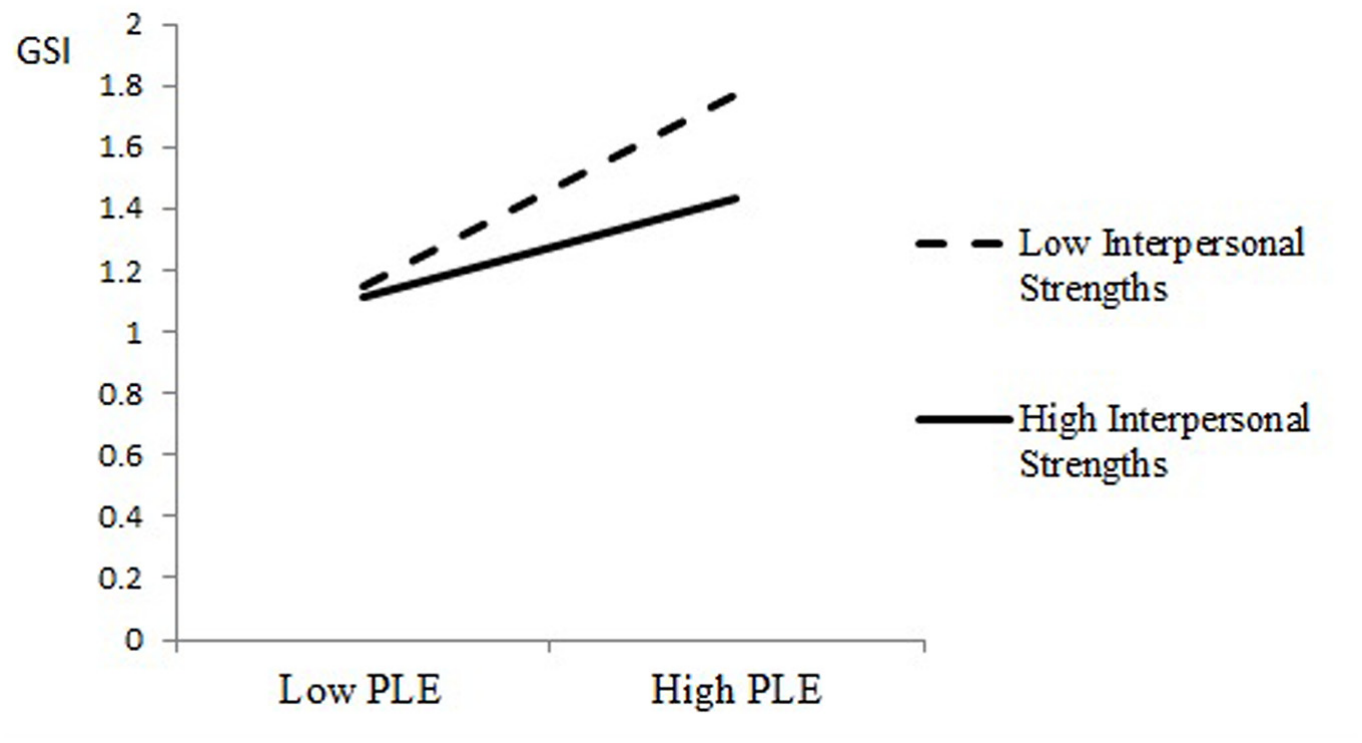
**General distress as a function of PLE exposure and interpersonal strengths**.

The main effect of PLE was moderated by an interaction between PLE exposure and interpersonal strengths on GSI levels (*b* = -0.11, *SE* = 0.03, *t* = 4.09, *p* < 0.001), as presented in **Figure 1.** To clarify the source of this interaction, we examined the GSI as a function of PLE exposure and the interpersonal strengths at 1 standard deviation above and below the mean (*M* = 1.36, *SD* = 0.81) (Cohen and Cohen, 1983). As seen in **Figure 1**, at low PLE exposure, there were no significant differences in GSI levels between participants with low interpersonal strengths (*M* = 1.15) and high interpersonal strengths (*M* = 1.11). However, at high PLE exposure, participants with low interpersonal strengths reported significantly higher GSI levels (*M* = 1.77) than participants with high interpersonal strengths (*M* = 1.43).

### Prediction of PTSD Symptoms by PLE Exposure and Strengths Factors

**FIGURE 2:**
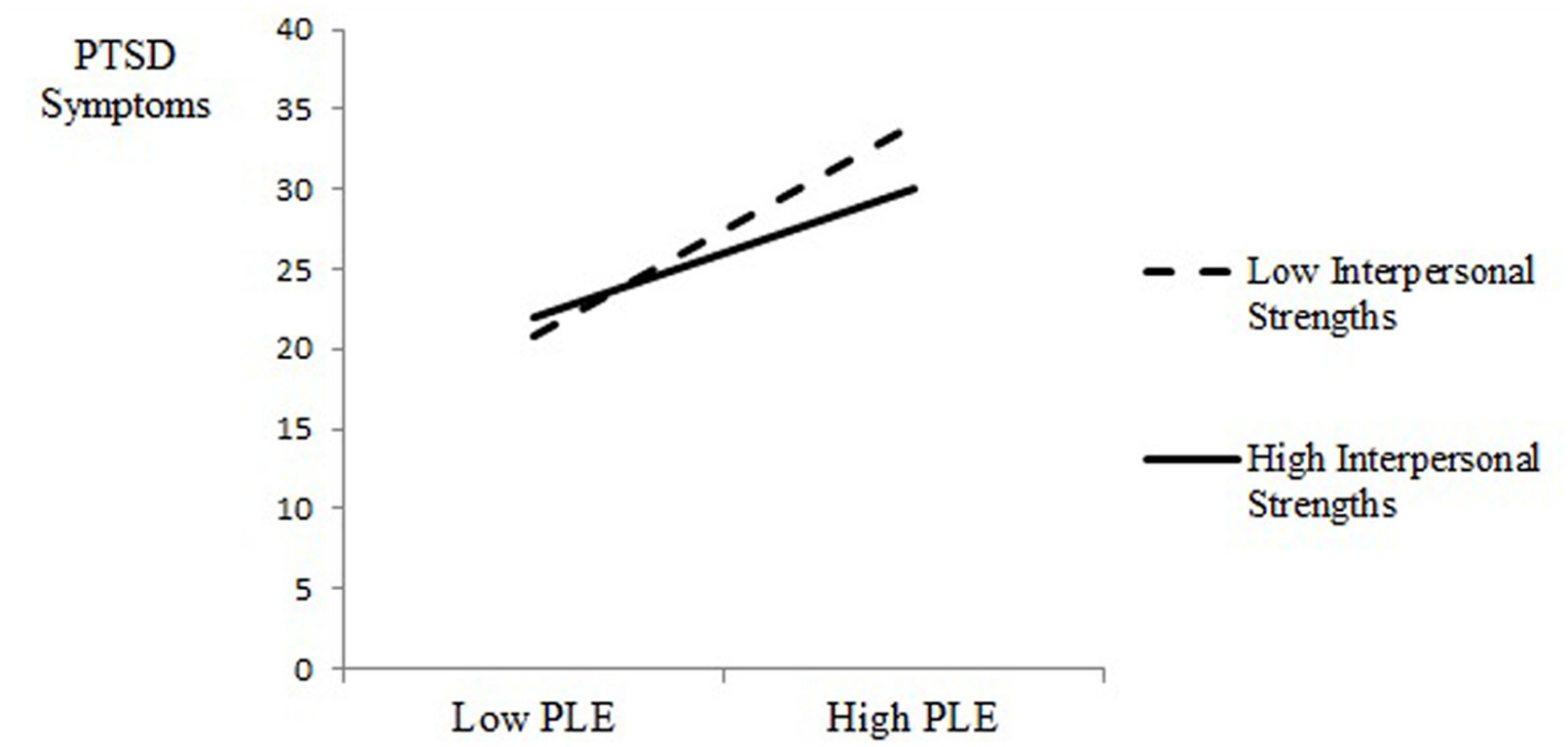
**Post-traumatic stress disorder (PTSD) symptoms as a function of PLE exposure and interpersonal strength**.

The regression analysis conducted on PTSD symptoms, *R* = 0.39, *R*^2^ = 0.15, *F*(6,1071) = 25.91, *p* < 0.001, revealed a significant effect for the temperance strengths (*b* = -0.09, *SE* = 0.03, *t* = 2.73, *p* = 0.006) and for PLE exposure (*b* = 0.39, *SE* = 0.03, *t* = 11.42, *p* = 0.001) on PTSD symptoms (see **Table 4**). The main effect of PLE exposure was moderated by an interaction between PLE and interpersonal strengths (*b* = -0.09, *SE* = 0.03, *t* = 2.67, *p* = 0.007). To clarify the source of this interaction, we examined PTSD symptoms as a function of PLE exposure and interpersonal strengths at 1 standard deviation above and below the mean (*M* = 26.51, *SD* = 14.51). As seen in **Figure 2**, at high PLE exposure, participants with low interpersonal strengths reported significantly higher PTSD symptoms (*M* = 34.14) than participants with high interpersonal strengths (*M* = 30.06). However, this effect was not obtained at low PLE exposure because there were no significant differences in PTSD symptoms between participants with low interpersonal strengths (*M* = 20.81) and high interpersonal strengths (*M* = 21.90).

## Discussion

The high levels of psychological distress, psychiatric symptoms and PTSD symptoms found among the adolescents in this study attest to the deleterious effects of growing up under protracted conflict and war. Strong correlations between PLEs exposure and these mental health symptoms confirm the dose-response relation between exposure to chronic conflict and outcomes (Braun-Lewensohn et al., 2009). Outcomes were not limited to PTSD but ranged across diverse symptoms and psychological difficulties including depression, anxiety, somatization, and post-traumatic stress. These severe effects to adolescents of growing up under threat and danger urge empirical research into those factors that can promote resilience and buffer negative outcomes.

The hypothesis predicting negative correlations between the strengths factors and the outcome variables was partially confirmed. For the GSI, there was a negative correlation with the temperance, transcendence and interpersonal strengths, while intellectual strengths were positively correlated with the GSI. Temperance strengths were also negatively correlated with PTSD symptoms.

These three factors appear to represent different mechanisms for coping with exposure to political violence. The transcendence factor, consisting of character strengths associated with positive framing of life circumstances, showed the highest moderation effect. This factor consists of strengths such as gratitude, religiousness, hope, zest, humor, and forgiveness. The common denominator of these strengths is mobilization of emotional processes that focus on the positive aspects of the experience, even in the face of a difficult reality (Fredrickson et al., 2003). The temperance factor was found to moderate both level of distress and post-traumatic symptoms. This factor consists of strengths such as self-regulation, authenticity, perseverance and prudence. As opposed to the transcendence factor, these strengths contain the essential element of regulation, both emotional and behavioral, and thus function as a moderator by preventing escalation of impulsive thoughts and behaviors during times of extreme stress (Cole et al., 2004).

The finding of a relation between the interpersonal strengths and lower levels of psychological distress concur with research evidence showing the beneficial effects of engaging in interpersonal relationships and mobilizing social support in conditions of war and armed conflict (Fremont, 2004). These findings can be summarized as showing that the intrapsychic and interpersonal character strengths facilitate lower levels of distress. Emotional and behavioral regulation (Eisenberg et al., 1998), interpersonal support (Betancourt and Khan, 2008) and sense of meaning and positive appraisal (Fernando, 2007), separately and together, can provide a base for coping with stressful life circumstances.

Interestingly, however, the intellectual strengths were correlated with higher levels of psychological distress. In other domains, such as research in interpersonal violence, the literature is mixed as to the relation between intellectual abilities and psychological symptoms, with findings along the entire range indicating that high intellectual ability may either increase stress symptoms or have no relation (Saltzman et al., 2006). This may be understood by emphasizing the distinction between the intellectual strengths and intellectual abilities. The intellectual strengths do not necessarily represent higher intelligence or information processing abilities. Rather, they represent motivational personality tendencies to seek and obtain knowledge through curiosity and love of learning. In contexts of exposure to war, terrorism and conflict, the search for knowledge and information may not necessarily be helpful in alleviating stress. To the contrary, some research has shown that high levels of media exposure to episodes of terrorism leads to increased anxiety and psychiatric symptoms (Slone and Shoshani, 2010). Similarly, high levels of consumption of news coverage after 9/11 were found to be associated with increased post-traumatic symptoms (Ahern et al., 2002). Thus, the need for knowledge and for satisfying curiosity may have a negative effect on mental health symptoms, especially when it entails exposure to scenes of horror. In addition, the strengths of fairness and appreciation of beauty, also delineated in the intellectual factor, could be asynchronous with conditions of war and armed conflict that frequently involve a sense of unfairness and exposure to the ugly side of human acts. Thus, the intellectual factor usually assumed to function as a resilience factor, could become a risk factor during war and hostilities. This proposition raises the issue of the context-dependent nature of the function served by the character strengths, which can vary from serving as resilience to risk factors depending on context and circumstance.

It is possible that it is not intellectual ability *per se* that aid in coping with trauma, such as high levels of attention to stimuli and constant information-seeking, but rather the capacity for regulating cognitions and attributing appropriate appraisals of the situation. The classic theory of coping according to Lazarus and Folkman (1984) defines adequate coping in terms of cognitive appraisals that enable management of specific internal or external demands that are taxing or that exceed the resources of the individual. This suggests that intellectual ability in and of itself is insufficient for coping with traumatic circumstances, but rather that the cognitive transformative process enables appropriate appraisals and alleviates stress.

The exploratory question examined whether the character strengths serve as resilience factors for exposure to chronic political violence. For both the GSI distress index and post-traumatic symptoms, interactions emerged between PLE exposure and the interpersonal strengths. At low PLE exposure, there were no significant differences in the GSI and PTSD symptoms for adolescents with high or low interpersonal strengths. However, with high exposure to political violence, adolescents with low interpersonal strengths reported significantly higher GSI and PTSD symptom levels than adolescents with high interpersonal strengths. In this study, individuals with stronger interpersonal strengths who were able to activate the ability for teamwork, love, social intelligence and bravery showed lower levels of psychological symptoms.

These findings imply that at high levels of political violence exposure, the remarkable ability to love and give, the ability to solicit supportive and comforting relationships, and the tendency for altruism and helping others, indeed serves as a moderating factor in the face of trauma. Interestingly, at times of great personal stress, it is the ability to be part of a loving and supportive interpersonal unit that serves the individual best.

This is so beyond the other character strengths for which there were no differences in GSI and PTSD symptoms at high or low political violence exposure. In other words, transcendence and temperance strengths functioned similarly for adolescents at high or low exposure. The main effects that emerged show that high levels of both these character strengths factors were related to less psychological distress and symptoms, both at low and high exposure.

This study had several limitations that should be addressed in further research. Data collection was based on self-report and should be supplemented with multi-informant reports. This is especially important in the case of character strengths that may be transparent to biased reporting. The character strengths inventory examines characterological traits that may not necessarily be played out in behavior. Comprehension of the resilience function of character strengths would be enhanced with additional inclusion of behavioral sampling methods such as structured observations and behavioral measures. The effects of prolonged political violence on the character of adolescents should ultimately be comprehensively examined by means of longitudinal study.

These findings of this study highlight the relevance of character development initiatives that aim to maximize individual personality resources in the face of difficult circumstances, particularly among high-risk adolescents. The literature has stressed the relation between character strengths and moral development, ethics and pro-social behavior. However, this study emphasizes that character strengths can and should be perceived additionally as resilience factors in highly stressful situations. This perspective should be continued in further research.

Growing up in a climate of protracted war, conflict and political violence can have an eroding effect on the personality of adolescents. It is the duty of society to explore ways to enhance resilience of adolescents exposed to these unfortunate circumstances that have increased worldwide.

## Conflict of Interest Statement

The authors declare that the research was conducted in the absence of any commercial or financial relationships that could be construed as a potential conflict of interest.
